# {4,4′,5,5′-Tetra­methyl-2,2′-[1,1′-(ethane-1,2-diyldinitrilo)diethyl­idyne]diphenolato}nickel(II)–methanol–chloro­form (1/1/1)

**DOI:** 10.1107/S1600536808024306

**Published:** 2008-08-06

**Authors:** Hoong-Kun Fun, Reza Kia

**Affiliations:** a X-ray Crystallography Unit, School of Physics, Universiti Sains Malaysia, 11800 USM, Penang, Malaysia

## Abstract

In the title compound, [Ni(C_22_H_26_N_2_O_2_)]·CH_3_OH·CHCl_3_, the Ni^II^ ion is in a slightly distorted square-planar geometry involving an N_2_O_2_ atom set of the tetra­dentate Schiff base ligand. The asymmetric unit contains one mol­ecule of the complex and one mol­ecule each of chloro­form and methanol. The methanol mol­ecule is hydrogen bonded to the phenolate O atoms. In the crystal structure, short inter­molecular distances between the centroids of six-membered chelate rings [3.7002 (9) Å] indicate the presence of π–π inter­actions, which link the mol­ecules into stacks along the *a* axis. In addition, there are Ni⋯Ni distances which are shorter than the sum of the van der Waals radii of two Ni atoms. The crystal structure is further stabilized by inter­molecular O—H⋯O and C—H⋯O hydrogen bonds, and weak inter­molecular C—H⋯π inter­actions linking mol­ecules into extended one-dimensional chains along the *c* axis.

## Related literature

For bond-length data, see Allen *et al.* (1987 ▶). For related structures see, for example: Clark *et al.* (1968 ▶, 1969 ▶, 1970 ▶). For applications and bioactivities see, for example: Elmali *et al.* (2000 ▶); Blower (1998 ▶); Granovski *et al.* (1993 ▶); Li & Chang (1991 ▶); Shahrokhian *et al.* (2000 ▶).
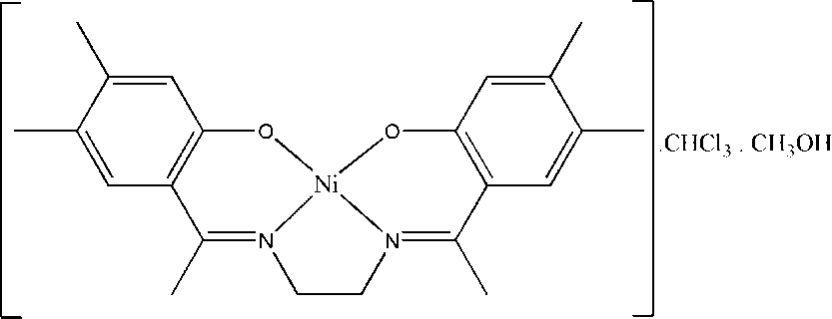


## Experimental

### 

#### Crystal data

[Ni(C_22_H_26_N_2_O_2_)]·CH_4_O·CHCl_3_
*M* *_r_* = 560.59
Triclinic, 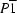

*a* = 7.5473 (1) Å
*b* = 12.3899 (2) Å
*c* = 14.2481 (2) Å
α = 75.949 (1)°
β = 83.761 (1)°
γ = 74.693 (1)°
*V* = 1245.21 (3) Å^3^
*Z* = 2
Mo *K*α radiation
μ = 1.13 mm^−1^
*T* = 100.0 (1) K
0.36 × 0.17 × 0.11 mm

#### Data collection

Bruker SMART APEXII CCD area-detector diffractometer
Absorption correction: multi-scan (*SADABS*; Bruker, 2005 ▶) *T*_min_ = 0.684, *T*_max_ = 0.882
29477 measured reflections
7348 independent reflections
5851 reflections with *I* > 2σ(*I*)
*R*_int_ = 0.038

#### Refinement

*R*[*F* ^2^ > 2σ(*F* ^2^)] = 0.040
*wR*(*F* ^2^) = 0.103
*S* = 1.04
7348 reflections
305 parameters
H-atom parameters constrained
Δρ_max_ = 0.71 e Å^−3^
Δρ_min_ = −0.80 e Å^−3^

### 

Data collection: *APEX2* (Bruker, 2005 ▶); cell refinement: *APEX2*; data reduction: *SAINT* (Bruker, 2005 ▶); program(s) used to solve structure: *SHELXTL* (Sheldrick, 2008 ▶); program(s) used to refine structure: *SHELXTL*; molecular graphics: *SHELXTL*; software used to prepare material for publication: *SHELXTL* and *PLATON* (Spek, 2003 ▶).

## Supplementary Material

Crystal structure: contains datablocks global, I. DOI: 10.1107/S1600536808024306/lh2659sup1.cif

Structure factors: contains datablocks I. DOI: 10.1107/S1600536808024306/lh2659Isup2.hkl

Additional supplementary materials:  crystallographic information; 3D view; checkCIF report

## Figures and Tables

**Table:** 

Ni1—O2	1.8276 (13)
Ni1—O1	1.8298 (13)
Ni1—N1	1.8534 (15)
Ni1—N2	1.8592 (16)

**Table:** 

Ni1⋯Ni1^i^	4.1276 (3)
Ni1⋯Ni1^ii^	4.5626 (3)

**Table:** 

O2—Ni1—O1	82.98 (6)
O2—Ni1—N1	177.05 (6)
O1—Ni1—N1	94.26 (6)
O2—Ni1—N2	93.94 (6)
O1—Ni1—N2	176.90 (6)
N1—Ni1—N2	88.82 (7)

Symmetry codes: (i) 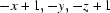; (ii) 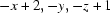.

**Table 2:** Hydrogen-bond geometry (Å, °)

*D*—H⋯*A*	*D*—H	H⋯*A*	*D*⋯*A*	*D*—H⋯*A*
O3—H1*O*3⋯O1	0.89	2.23	2.980 (2)	142
O3—H1*O*3⋯O2	0.89	2.10	2.901 (2)	149
C23—H23*A*⋯O3	0.98	2.10	2.974 (3)	148
C9—H9*A*⋯*Cg*1^ii^	0.97	2.47	3.404 (2)	162
C20—H20*A*⋯*Cg*2^ii^	0.96	2.94	3.801 (2)	150
C21—H21*B*⋯*Cg*3^iii^	0.96	2.82	3.691 (2)	152

Symmetry codes: (ii) 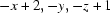; (iii) 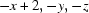. *Cg*1, *Cg*2 and *Cg*3 are the centroids of the Ni1/O1/C1/C6/C7/N1, C1–C6, and C11–C16 rings, respectively.

**Fig. 1.**
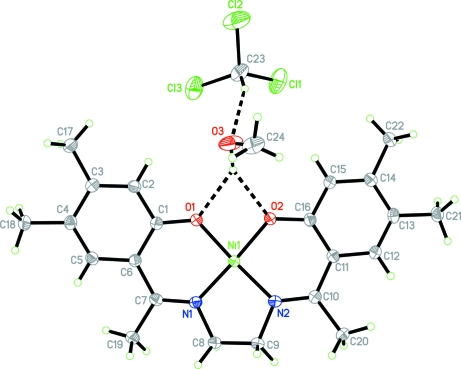
The molecular structure of the title compound, showing 50% probability displacement ellipsoids and the atomic numbering. Intermolecular hydrogen bonds are drawn as dashed lines.

**Fig. 2.**
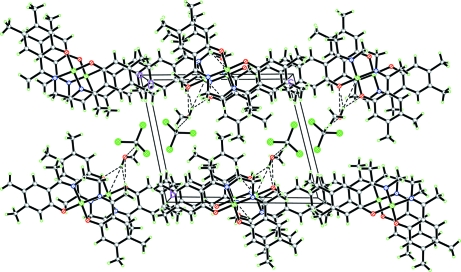
The crystal packing of (I), showing stacks viewed down the a axis. Intermolecular interactions are drawn as dashed lines.

## supplementary crystallographic information

### Comment

Schiff base complexes are some of the most important stereochemical models in transition metal coordination chemistry, with their ease of preparation and structural variations (Granovski *et al.*, 1993). Metal derivatives of Schiff bases have been studied extensively, and copper(II) and Ni(II) complexes play a major role in both synthetic and structural research (Elmali *et al.*, 2000; Blower, 1998; Granovski *et al.*, 1993; Li & Chang, 1991; Shahrokhian *et al.*, 2000). Tetradentate Schiff base metal complexes may form *trans* or *cis* planar or tetrahedral structures (Elmali *et al.*, 2000).

In the title compound (I, Fig. 1), the Ni^II^ ion shows a sligthly distorted square-planar geometry which is coordinated by two imine N atoms and two phenol O atoms of the tetradentate Schiff base ligand. The bond lenghts and angles are within the normal ranges (Allen *et al.*, 1987). The asymmetric unit of the compound contains one molecule of the complex, and a molecule each of the chloroform and the methanol solvents. The methanol molecule is H-bonded to the phenolato oxygen atoms of the complex. Atoms C8 and C9 are significantly out of the plane, as indicated by the torsion angle N1–C8–C9–N2, which is -31.8 (2)°. The dihedral angle between two benzene rings is 11.11 (9)°. The planar molecules are stacked into columns along the *a* axis, with Ni···Ni [(i) 1 - *x*, -*y*, 1 - *z* and (ii) 2 - *x*, -*y*, 1 - *z*] separations of 4.1276 (3) to 4.5626 (3) Å are shorter than the sum of the van der Waals radii of two Ni atoms (4.60 Å). The short intermolecular distances between the centroids of six-membered rings [3.7002 (9) Å] prove an existence of π-π interactions, which link the molecules into one-dimensional extended chains along the *a* axis (Fig. 2). The crystal packing is further stabilized by intermolecular O—H···O, C—H···O hydrogen bonds and weak intermolecular C—H···π interactions..

### Experimental

A chloroform solution (40 ml) of the ligand (1 mmol, 354 mg) was added to a methanol solution (20) of NiCl_2_.6H_2_O (1.05 mmol, 237 mg). The mixture was refluxed for 30 min and then filtered. After keeping the filtrate in air for 4 d, pink block-shaped crystals were formed at the bottom of the vessel on slow evaporation of the solvent.

### Refinement

The H-atom attached to O3 was located in a difference Fourier map and refined as riding with the parent atom with an isotropic thermal parameter 1.5 times that of the parent atom. The rest of the hydrogen atoms were positioned geometrically [C—H = 0.93–97 Å] and refind using a riding model. A rotating-group model was used for the methyl groups.

### Crystal data

**Table:**

[Ni(C_22_H_26_N_2_O_2_)]·CH_4_O·CHCl_3_	*Z* = 2
*M**_r_* = 560.59	*F*_000_ = 584
Triclinic, *P*1	*D*_x_ = 1.495 Mg m^−^^3^
Hall symbol: -P 1	Mo *K*α radiation λ = 0.71073 Å
*a* = 7.5473 (1) Å	Cell parameters from 7819 reflections
*b* = 12.3899 (2) Å	θ = 2.5–29.3º
*c* = 14.2481 (2) Å	µ = 1.13 mm^−^^1^
α = 75.949 (1)º	*T* = 100.0 (1) K
β = 83.761 (1)º	Block, pink
γ = 74.693 (1)º	0.36 × 0.17 × 0.11 mm
*V* = 1245.21 (3) Å^3^	

### Data collection

**Table:**

Bruker SMART APEXII CCD area-detector diffractometer	7348 independent reflections
Radiation source: fine-focus sealed tube	5851 reflections with *I* > 2σ(*I*)
Monochromator: graphite	*R*_int_ = 0.038
*T* = 100.0(1) K	θ_max_ = 30.2º
φ and ω scans	θ_min_ = 2.0º
Absorption correction: multi-scan(SADABS; Bruker, 2005)	*h* = −10→10
*T*_min_ = 0.684, *T*_max_ = 0.882	*k* = −16→17
29477 measured reflections	*l* = −20→20

### Refinement

**Table:**

Refinement on *F*^2^	Secondary atom site location: difference Fourier map
Least-squares matrix: full	Hydrogen site location: inferred from neighbouring sites
*R*[*F*^2^ > 2σ(*F*^2^)] = 0.040	H-atom parameters constrained
*wR*(*F*^2^) = 0.103	*w* = 1/[σ^2^(*F*_o_^2^) + (0.0463*P*)^2^ + 0.6625*P*] where *P* = (*F*_o_^2^ + 2*F*_c_^2^)/3
*S* = 1.04	(Δ/σ)_max_ = 0.001
7348 reflections	Δρ_max_ = 0.71 e Å^−^^3^
305 parameters	Δρ_min_ = −0.80 e Å^−^^3^
Primary atom site location: structure-invariant direct methods	Extinction correction: none

### Special details

**Table:**

Experimental. The low-temperature data was collected with the Oxford Cyrosystem Cobra low-temperature attachment.
Geometry. All e.s.d.'s (except the e.s.d. in the dihedral angle between two l.s. planes) are estimated using the full covariance matrix. The cell e.s.d.'s are taken into account individually in the estimation of e.s.d.'s in distances, angles and torsion angles; correlations between e.s.d.'s in cell parameters are only used when they are defined by crystal symmetry. An approximate (isotropic) treatment of cell e.s.d.'s is used for estimating e.s.d.'s involving l.s. planes.
Refinement. Refinement of *F*^2^ against ALL reflections. The weighted *R*-factor *wR* and goodness of fit *S* are based on *F*^2^, conventional *R*-factors *R* are based on *F*, with *F* set to zero for negative *F*^2^. The threshold expression of *F*^2^ > σ(*F*^2^) is used only for calculating *R*-factors(gt) *etc*. and is not relevant to the choice of reflections for refinement. *R*-factors based on *F*^2^ are statistically about twice as large as those based on *F*, and *R*- factors based on ALL data will be even larger.

### Fractional atomic coordinates and isotropic or equivalent isotropic displacement parameters (Å^2^)

**Table:**

	*x*	*y*	*z*	*U*_iso_*/*U*_eq_	
Ni1	0.73830 (3)	0.02552 (2)	0.422240 (16)	0.01582 (7)	
O1	0.59273 (18)	0.16632 (11)	0.43248 (9)	0.0194 (3)	
O2	0.72158 (19)	0.08848 (11)	0.29250 (9)	0.0221 (3)	
N1	0.7520 (2)	−0.03073 (13)	0.55493 (11)	0.0168 (3)	
N2	0.8874 (2)	−0.11446 (13)	0.40463 (11)	0.0169 (3)	
C1	0.5369 (2)	0.20434 (16)	0.51176 (13)	0.0173 (3)	
C2	0.4308 (3)	0.31894 (16)	0.49954 (13)	0.0192 (4)	
H2A	0.4036	0.3612	0.4371	0.023*	
C3	0.3656 (3)	0.37099 (16)	0.57621 (14)	0.0198 (4)	
C4	0.4074 (2)	0.30783 (17)	0.67159 (13)	0.0199 (4)	
C5	0.5071 (2)	0.19556 (17)	0.68422 (13)	0.0195 (4)	
H5A	0.5317	0.1539	0.7471	0.023*	
C6	0.5749 (2)	0.13932 (16)	0.60716 (13)	0.0175 (3)	
C7	0.6784 (2)	0.01988 (16)	0.62568 (13)	0.0179 (3)	
C8	0.8448 (3)	−0.15402 (16)	0.58025 (13)	0.0194 (4)	
H8A	0.7540	−0.1991	0.5959	0.023*	
H8B	0.9172	−0.1700	0.6365	0.023*	
C9	0.9685 (3)	−0.18622 (16)	0.49521 (13)	0.0193 (4)	
H9A	1.0894	−0.1747	0.4995	0.023*	
H9B	0.9822	−0.2666	0.4963	0.023*	
C10	0.9362 (2)	−0.15019 (16)	0.32411 (13)	0.0176 (3)	
C11	0.8610 (2)	−0.08203 (16)	0.23219 (13)	0.0175 (3)	
C12	0.8869 (2)	−0.13058 (16)	0.14945 (13)	0.0187 (4)	
H12A	0.9530	−0.2066	0.1556	0.022*	
C14	0.7214 (2)	0.04489 (17)	0.05063 (13)	0.0187 (4)	
C15	0.6937 (3)	0.09445 (16)	0.12986 (13)	0.0195 (4)	
H15A	0.6291	0.1709	0.1224	0.023*	
C16	0.7598 (3)	0.03323 (16)	0.22177 (13)	0.0185 (4)	
C17	0.2529 (3)	0.49346 (17)	0.55849 (15)	0.0256 (4)	
H17A	0.2465	0.5250	0.4900	0.038*	
H17B	0.3094	0.5377	0.5873	0.038*	
H17C	0.1311	0.4957	0.5869	0.038*	
C18	0.3461 (3)	0.36271 (18)	0.75716 (14)	0.0263 (4)	
H18A	0.3831	0.3070	0.8158	0.039*	
H18B	0.2147	0.3904	0.7593	0.039*	
H18C	0.4016	0.4257	0.7508	0.039*	
C19	0.7027 (3)	−0.04867 (18)	0.72865 (14)	0.0252 (4)	
H19A	0.6730	−0.1209	0.7348	0.038*	
H19B	0.6226	−0.0067	0.7717	0.038*	
H19C	0.8280	−0.0621	0.7449	0.038*	
C20	1.0714 (3)	−0.26399 (16)	0.32619 (14)	0.0210 (4)	
H20A	1.1643	−0.2748	0.3709	0.031*	
H20B	1.1278	−0.2655	0.2626	0.031*	
H20C	1.0084	−0.3244	0.3464	0.031*	
C21	0.8500 (3)	−0.12856 (18)	−0.02354 (14)	0.0242 (4)	
H21A	0.9156	−0.2074	−0.0029	0.036*	
H21B	0.9204	−0.0900	−0.0746	0.036*	
H21C	0.7333	−0.1251	−0.0467	0.036*	
C22	0.6465 (3)	0.11405 (17)	−0.04537 (13)	0.0224 (4)	
H22A	0.5839	0.1905	−0.0394	0.034*	
H22B	0.5618	0.0786	−0.0646	0.034*	
H22C	0.7458	0.1172	−0.0933	0.034*	
Cl1	0.24264 (10)	0.37079 (6)	0.07683 (6)	0.05382 (19)	
Cl2	0.28123 (13)	0.59955 (6)	0.06488 (7)	0.0640 (2)	
C13	0.8193 (2)	−0.07080 (17)	0.06089 (13)	0.0197 (4)	
Cl3	0.18264 (9)	0.46457 (7)	0.24800 (5)	0.05064 (18)	
C23	0.3093 (3)	0.4605 (2)	0.13651 (18)	0.0350 (5)	
H23A	0.4398	0.4293	0.1499	0.042*	
O3	0.6398 (2)	0.33720 (14)	0.25019 (13)	0.0431 (4)	
H1O3	0.6568	0.2655	0.2858	0.065*	
C24	0.8172 (3)	0.3341 (2)	0.20835 (19)	0.0389 (5)	
H24A	0.8137	0.4002	0.1561	0.058*	
H24B	0.8625	0.2657	0.1839	0.058*	
H24C	0.8971	0.3343	0.2563	0.058*	

### Atomic displacement parameters (Å^2^)

**Table:**

	*U*^11^	*U*^22^	*U*^33^	*U*^12^	*U*^13^	*U*^23^
Ni1	0.01784 (12)	0.01479 (12)	0.01401 (11)	−0.00247 (8)	−0.00203 (8)	−0.00282 (8)
O1	0.0229 (7)	0.0177 (6)	0.0157 (6)	−0.0012 (5)	−0.0018 (5)	−0.0041 (5)
O2	0.0332 (8)	0.0167 (7)	0.0146 (6)	−0.0019 (6)	−0.0026 (5)	−0.0038 (5)
N1	0.0164 (7)	0.0154 (7)	0.0180 (7)	−0.0030 (6)	−0.0023 (6)	−0.0030 (6)
N2	0.0164 (7)	0.0164 (7)	0.0171 (7)	−0.0036 (6)	−0.0023 (5)	−0.0019 (6)
C1	0.0160 (8)	0.0185 (9)	0.0183 (8)	−0.0049 (7)	−0.0010 (6)	−0.0050 (7)
C2	0.0204 (9)	0.0185 (9)	0.0178 (8)	−0.0042 (7)	−0.0025 (7)	−0.0022 (7)
C3	0.0179 (8)	0.0187 (9)	0.0231 (9)	−0.0053 (7)	−0.0001 (7)	−0.0051 (7)
C4	0.0172 (9)	0.0225 (9)	0.0205 (9)	−0.0041 (7)	0.0005 (7)	−0.0075 (7)
C5	0.0173 (8)	0.0230 (9)	0.0174 (8)	−0.0036 (7)	−0.0010 (6)	−0.0047 (7)
C6	0.0163 (8)	0.0184 (9)	0.0179 (8)	−0.0039 (7)	−0.0010 (6)	−0.0043 (7)
C7	0.0154 (8)	0.0203 (9)	0.0172 (8)	−0.0046 (7)	−0.0011 (6)	−0.0022 (7)
C8	0.0217 (9)	0.0168 (9)	0.0179 (8)	−0.0025 (7)	−0.0034 (7)	−0.0017 (7)
C9	0.0207 (9)	0.0181 (9)	0.0172 (8)	−0.0019 (7)	−0.0037 (7)	−0.0023 (7)
C10	0.0166 (8)	0.0176 (9)	0.0195 (8)	−0.0055 (7)	−0.0005 (6)	−0.0047 (7)
C11	0.0190 (9)	0.0177 (9)	0.0170 (8)	−0.0065 (7)	−0.0009 (6)	−0.0039 (7)
C12	0.0184 (9)	0.0181 (9)	0.0202 (9)	−0.0041 (7)	−0.0002 (7)	−0.0065 (7)
C14	0.0161 (8)	0.0227 (9)	0.0178 (8)	−0.0067 (7)	−0.0001 (6)	−0.0035 (7)
C15	0.0215 (9)	0.0177 (9)	0.0187 (8)	−0.0033 (7)	−0.0007 (7)	−0.0050 (7)
C16	0.0205 (9)	0.0187 (9)	0.0170 (8)	−0.0061 (7)	0.0006 (6)	−0.0046 (7)
C17	0.0286 (10)	0.0197 (10)	0.0259 (10)	−0.0024 (8)	0.0010 (8)	−0.0053 (8)
C18	0.0299 (11)	0.0255 (10)	0.0226 (9)	−0.0013 (8)	−0.0010 (8)	−0.0100 (8)
C19	0.0293 (10)	0.0242 (10)	0.0171 (9)	−0.0005 (8)	−0.0014 (7)	−0.0019 (7)
C20	0.0204 (9)	0.0182 (9)	0.0228 (9)	−0.0007 (7)	−0.0011 (7)	−0.0061 (7)
C21	0.0245 (10)	0.0292 (11)	0.0207 (9)	−0.0048 (8)	−0.0021 (7)	−0.0107 (8)
C22	0.0237 (9)	0.0260 (10)	0.0179 (9)	−0.0068 (8)	−0.0010 (7)	−0.0048 (7)
Cl1	0.0519 (4)	0.0516 (4)	0.0728 (5)	−0.0196 (3)	0.0068 (3)	−0.0386 (4)
Cl2	0.0768 (6)	0.0307 (4)	0.0800 (6)	−0.0115 (3)	−0.0152 (4)	−0.0009 (4)
C13	0.0176 (8)	0.0250 (10)	0.0189 (8)	−0.0074 (7)	0.0020 (7)	−0.0085 (7)
Cl3	0.0402 (4)	0.0571 (4)	0.0579 (4)	−0.0051 (3)	0.0034 (3)	−0.0290 (3)
C23	0.0320 (12)	0.0268 (11)	0.0483 (14)	−0.0049 (9)	−0.0056 (10)	−0.0131 (10)
O3	0.0401 (10)	0.0246 (8)	0.0521 (11)	−0.0019 (7)	−0.0001 (8)	0.0073 (7)
C24	0.0369 (13)	0.0270 (12)	0.0498 (15)	−0.0053 (10)	−0.0087 (11)	−0.0028 (11)

### Geometric parameters (Å, °)

**Table:**

Ni1—O2	1.8276 (13)	C14—C15	1.383 (3)
Ni1—O1	1.8298 (13)	C14—C13	1.409 (3)
Ni1—N1	1.8534 (15)	C14—C22	1.506 (3)
Ni1—N2	1.8592 (16)	C15—C16	1.414 (3)
O1—C1	1.314 (2)	C15—H15A	0.9300
O2—C16	1.317 (2)	C17—H17A	0.9600
N1—C7	1.311 (2)	C17—H17B	0.9600
N1—C8	1.475 (2)	C17—H17C	0.9600
N2—C10	1.310 (2)	C18—H18A	0.9600
N2—C9	1.469 (2)	C18—H18B	0.9600
C1—C2	1.413 (3)	C18—H18C	0.9600
C1—C6	1.418 (2)	C19—H19A	0.9600
C2—C3	1.382 (3)	C19—H19B	0.9600
C2—H2A	0.9300	C19—H19C	0.9600
C3—C4	1.416 (3)	C20—H20A	0.9600
C3—C17	1.506 (3)	C20—H20B	0.9600
C4—C5	1.374 (3)	C20—H20C	0.9600
C4—C18	1.509 (3)	C21—C13	1.511 (3)
C5—C6	1.419 (3)	C21—H21A	0.9600
C5—H5A	0.9300	C21—H21B	0.9600
C6—C7	1.454 (3)	C21—H21C	0.9600
C7—C19	1.510 (2)	C22—H22A	0.9600
C8—C9	1.514 (3)	C22—H22B	0.9600
C8—H8A	0.9700	C22—H22C	0.9600
C8—H8B	0.9700	Cl1—C23	1.749 (2)
C9—H9A	0.9700	Cl2—C23	1.748 (3)
C9—H9B	0.9700	Cl3—C23	1.767 (3)
C10—C11	1.457 (3)	C23—H23A	0.9800
C10—C20	1.502 (3)	O3—C24	1.400 (3)
C11—C16	1.411 (3)	O3—H1O3	0.8931
C11—C12	1.422 (2)	C24—H24A	0.9600
C12—C13	1.374 (3)	C24—H24B	0.9600
C12—H12A	0.9300	C24—H24C	0.9600
			
Ni1···Ni1^i^	4.1276 (3)	Ni1···Ni1^ii^	4.5626 (3)
			
O2—Ni1—O1	82.98 (6)	C14—C15—C16	122.47 (18)
O2—Ni1—N1	177.05 (6)	C14—C15—H15A	118.8
O1—Ni1—N1	94.26 (6)	C16—C15—H15A	118.8
O2—Ni1—N2	93.94 (6)	O2—C16—C11	124.29 (16)
O1—Ni1—N2	176.90 (6)	O2—C16—C15	117.16 (17)
N1—Ni1—N2	88.82 (7)	C11—C16—C15	118.55 (17)
C1—O1—Ni1	127.77 (12)	C3—C17—H17A	109.5
C16—O2—Ni1	126.95 (12)	C3—C17—H17B	109.5
C7—N1—C8	117.97 (15)	H17A—C17—H17B	109.5
C7—N1—Ni1	129.48 (13)	C3—C17—H17C	109.5
C8—N1—Ni1	112.23 (11)	H17A—C17—H17C	109.5
C10—N2—C9	119.07 (16)	H17B—C17—H17C	109.5
C10—N2—Ni1	128.89 (13)	C4—C18—H18A	109.5
C9—N2—Ni1	111.81 (12)	C4—C18—H18B	109.5
O1—C1—C2	116.60 (16)	H18A—C18—H18B	109.5
O1—C1—C6	124.99 (17)	C4—C18—H18C	109.5
C2—C1—C6	118.41 (16)	H18A—C18—H18C	109.5
C3—C2—C1	122.95 (17)	H18B—C18—H18C	109.5
C3—C2—H2A	118.5	C7—C19—H19A	109.5
C1—C2—H2A	118.5	C7—C19—H19B	109.5
C2—C3—C4	119.06 (18)	H19A—C19—H19B	109.5
C2—C3—C17	120.45 (17)	C7—C19—H19C	109.5
C4—C3—C17	120.50 (17)	H19A—C19—H19C	109.5
C5—C4—C3	118.32 (17)	H19B—C19—H19C	109.5
C5—C4—C18	120.77 (17)	C10—C20—H20A	109.5
C3—C4—C18	120.90 (17)	C10—C20—H20B	109.5
C4—C5—C6	124.01 (17)	H20A—C20—H20B	109.5
C4—C5—H5A	118.0	C10—C20—H20C	109.5
C6—C5—H5A	118.0	H20A—C20—H20C	109.5
C1—C6—C5	117.21 (17)	H20B—C20—H20C	109.5
C1—C6—C7	121.60 (16)	C13—C21—H21A	109.5
C5—C6—C7	121.19 (16)	C13—C21—H21B	109.5
N1—C7—C6	121.70 (16)	H21A—C21—H21B	109.5
N1—C7—C19	118.56 (17)	C13—C21—H21C	109.5
C6—C7—C19	119.73 (16)	H21A—C21—H21C	109.5
N1—C8—C9	109.21 (15)	H21B—C21—H21C	109.5
N1—C8—H8A	109.8	C14—C22—H22A	109.5
C9—C8—H8A	109.8	C14—C22—H22B	109.5
N1—C8—H8B	109.8	H22A—C22—H22B	109.5
C9—C8—H8B	109.8	C14—C22—H22C	109.5
H8A—C8—H8B	108.3	H22A—C22—H22C	109.5
N2—C9—C8	109.27 (15)	H22B—C22—H22C	109.5
N2—C9—H9A	109.8	C12—C13—C14	118.70 (17)
C8—C9—H9A	109.8	C12—C13—C21	120.37 (18)
N2—C9—H9B	109.8	C14—C13—C21	120.92 (17)
C8—C9—H9B	109.8	Cl2—C23—Cl1	111.33 (14)
H9A—C9—H9B	108.3	Cl2—C23—Cl3	109.86 (13)
N2—C10—C11	121.32 (17)	Cl1—C23—Cl3	110.63 (13)
N2—C10—C20	119.63 (16)	Cl2—C23—H23A	108.3
C11—C10—C20	119.06 (16)	Cl1—C23—H23A	108.3
C16—C11—C12	117.64 (16)	Cl3—C23—H23A	108.3
C16—C11—C10	121.81 (16)	C24—O3—H1O3	100.3
C12—C11—C10	120.55 (17)	O3—C24—H24A	109.5
C13—C12—C11	123.29 (18)	O3—C24—H24B	109.5
C13—C12—H12A	118.4	H24A—C24—H24B	109.5
C11—C12—H12A	118.4	O3—C24—H24C	109.5
C15—C14—C13	119.34 (17)	H24A—C24—H24C	109.5
C15—C14—C22	120.14 (18)	H24B—C24—H24C	109.5
C13—C14—C22	120.52 (17)		
			
O2—Ni1—O1—C1	−175.67 (15)	C5—C6—C7—N1	−175.42 (17)
N1—Ni1—O1—C1	3.28 (15)	C1—C6—C7—C19	−175.21 (17)
O1—Ni1—O2—C16	−163.17 (16)	C5—C6—C7—C19	4.6 (3)
N2—Ni1—O2—C16	17.21 (16)	C7—N1—C8—C9	−162.36 (16)
O1—Ni1—N1—C7	−0.21 (17)	Ni1—N1—C8—C9	23.57 (18)
N2—Ni1—N1—C7	179.36 (16)	C10—N2—C9—C8	−158.37 (16)
O1—Ni1—N1—C8	173.00 (12)	Ni1—N2—C9—C8	26.54 (18)
N2—Ni1—N1—C8	−7.43 (12)	N1—C8—C9—N2	−31.8 (2)
O2—Ni1—N2—C10	−6.81 (16)	C9—N2—C10—C11	−179.55 (15)
N1—Ni1—N2—C10	174.26 (16)	Ni1—N2—C10—C11	−5.4 (3)
O2—Ni1—N2—C9	167.68 (12)	C9—N2—C10—C20	0.9 (2)
N1—Ni1—N2—C9	−11.26 (12)	Ni1—N2—C10—C20	175.00 (12)
Ni1—O1—C1—C2	177.47 (12)	N2—C10—C11—C16	11.8 (3)
Ni1—O1—C1—C6	−2.7 (3)	C20—C10—C11—C16	−168.63 (16)
O1—C1—C2—C3	−178.80 (16)	N2—C10—C11—C12	−167.88 (16)
C6—C1—C2—C3	1.3 (3)	C20—C10—C11—C12	11.7 (2)
C1—C2—C3—C4	0.5 (3)	C16—C11—C12—C13	0.3 (3)
C1—C2—C3—C17	−179.93 (17)	C10—C11—C12—C13	179.94 (17)
C2—C3—C4—C5	−1.9 (3)	C13—C14—C15—C16	−0.1 (3)
C17—C3—C4—C5	178.57 (17)	C22—C14—C15—C16	179.49 (17)
C2—C3—C4—C18	177.35 (17)	Ni1—O2—C16—C11	−15.6 (3)
C17—C3—C4—C18	−2.2 (3)	Ni1—O2—C16—C15	164.22 (13)
C3—C4—C5—C6	1.5 (3)	C12—C11—C16—O2	178.46 (16)
C18—C4—C5—C6	−177.74 (17)	C10—C11—C16—O2	−1.2 (3)
O1—C1—C6—C5	178.44 (16)	C12—C11—C16—C15	−1.4 (3)
C2—C1—C6—C5	−1.7 (2)	C10—C11—C16—C15	178.98 (16)
O1—C1—C6—C7	−1.8 (3)	C14—C15—C16—O2	−178.51 (17)
C2—C1—C6—C7	178.11 (16)	C14—C15—C16—C11	1.3 (3)
C4—C5—C6—C1	0.3 (3)	C11—C12—C13—C14	0.9 (3)
C4—C5—C6—C7	−179.48 (17)	C11—C12—C13—C21	−179.10 (17)
C8—N1—C7—C6	−176.32 (15)	C15—C14—C13—C12	−1.0 (3)
Ni1—N1—C7—C6	−3.4 (3)	C22—C14—C13—C12	179.41 (16)
C8—N1—C7—C19	3.7 (2)	C15—C14—C13—C21	179.03 (17)
Ni1—N1—C7—C19	176.56 (13)	C22—C14—C13—C21	−0.6 (3)
C1—C6—C7—N1	4.8 (3)		

Symmetry codes: (i) −*x*+1, −*y*, −*z*+1; (ii) −*x*+2, −*y*, −*z*+1.

### Hydrogen-bond geometry (Å, °)

**Table:**

*D*—H···*A*	*D*—H	H···*A*	*D*···*A*	*D*—H···*A*
O3—H1O3···O1	0.89	2.23	2.980 (2)	142
O3—H1O3···O2	0.89	2.10	2.901 (2)	149
C23—H23A···O3	0.98	2.10	2.974 (3)	148
C9—H9A···Cg1^ii^	0.97	2.47	3.404 (2)	162
C20—H20A···Cg2^ii^	0.96	2.94	3.801 (2)	150
C21—H21B···Cg3^iii^	0.96	2.82	3.691 (2)	152

Symmetry codes: (ii) −*x*+2, −*y*, −*z*+1; (iii) −*x*+2, −*y*, −*z*.
